# Identification of Chronic Heart Failure Patients with a High 12-Month Mortality Risk Using Biomarkers Including Plasma C-Terminal Pro-Endothelin-1

**DOI:** 10.1371/journal.pone.0014506

**Published:** 2011-01-17

**Authors:** Ewa A. Jankowska, Gerasimos S. Filippatos, Stephan von Haehling, Jana Papassotiriou, Nils G. Morgenthaler, Mariantonietta Cicoira, Joerg C. Schefold, Piotr Rozentryt, Beata Ponikowska, Wolfram Doehner, Waldemar Banasiak, Oliver Hartmann, Joachim Struck, Andreas Bergmann, Stefan D. Anker, Piotr Ponikowski

**Affiliations:** 1 Department of Heart Diseases, Wroclaw Military Hospital, Wroclaw, Poland; 2 Cardiology Department, Military Hospital, Wroclaw, Poland; 3 Department of Cardiology, Atticon University Hospital, Athens, Greece; 4 Division of Applied Cachexia Research, Department of Cardiology, Charité Medical School, Berlin, Germany; 5 Department of Clinical Cardiology, National Heart and Lung Institute, Imperial College London, London, United Kingdom; 6 Research Department, B.R.A.H.M.S. AG, Biotechnology Centre Hennigsdorf/Berlin, Hennigsdorf, Germany; 7 Department of Biomedical and Surgical Sciences, Section of Cardiology, University of Verona, Verona, Italy; 8 Department of Nephrology and Intensive Care Medicine, Charité Medical School, Berlin, Germany; 9 Third Department of Cardiology, Silesian Center for Heart Disease, Zabrze, Poland; 10 Physiology Department, Wroclaw Medical University, Wroclaw, Poland; 11 Center for Stroke Research Berlin, Charité Medical School, Berlin, Germany; 12 Centre for Clinical and Basic Research, IRCCS San Raffaele, Rome, Italy; University of Padova, Italy

## Abstract

**Objectives:**

We hypothesised that assessment of plasma C-terminal pro-endothelin-1 (CT-proET-1), a stable endothelin-1 precursor fragment, is of prognostic value in patients with chronic heart failure (CHF), beyond other prognosticators, including N-terminal pro-B-type natriuretic peptide (NT-proBNP).

**Methods:**

We examined 491 patients with systolic CHF (age: 63±11 years, 91% men, New York Heart Association [NYHA] class [I/II/III/IV]: 9%/45%/38%/8%, 69% ischemic etiology). Plasma CT-proET-1 was detected using a chemiluminescence immunoassay.

**Results:**

Increasing CT-proET-1 was a predictor of increased cardiovascular mortality at 12-months of follow-up (standardized hazard ratio 1.42, 95% confidence interval [CI] 1.04–1.95, p = 0.03) after adjusting for NT-proBNP, left ventricular ejection fraction (LVEF), age, creatinine, NYHA class. In receiver operating characteristic curve analysis, areas under curve for 12-month follow-up were similar for CT-proET-1 and NT-proBNP (p = 0.40). Both NT-proBNP and CT-proET-1 added prognostic value to a base model that included LVEF, age, creatinine, and NYHA class. Adding CT-proET-1 to the base model had stronger prognostic power (p<0.01) than adding NT-proBNP (p<0.01). Adding CT-proET-1 to NT-proBNP in this model yielded further prognostic information (p = 0.02).

**Conclusions:**

Plasma CT-proET-1 constitutes a novel predictor of increased 12-month cardiovascular mortality in patients with CHF. High CT-proET-1 together with high NT-proBNP enable to identify patients with CHF and particularly unfavourable outcomes.

## Introduction

Endothelin-1 (ET-1) is a potent vasoconstrictor peptide secreted mainly by endothelial cells [1], [2]. It is also produced by other cells associated with cardiovascular homeostasis such as smooth muscle cells, cardiomyocytes, and macrophages [1], [2]. Activation of the ET-1 system is involved in the pathogenesis of several cardiovascular diseases, including hypertension, coronary artery disease, and chronic heart failure (CHF) [1], [2].

It is presumed that the assessment of circulating ET-1 might represent a marker of disease progression and prognosis in patients with CHF [3], [4]. This issue is of a particular clinical importance, as the prognostic evaluation in this group of patients still remains a challenge. The precise identification of patients with CHF and the highest risk of death and/or disease progression would enable clinicians to intensify both pharmacotherapy and device-based management early in the course of the disease. In recent years, a huge emphasis has been put on neurohumoral mediators as potential prognostic markers in patients with CHF [5], [6]. Over the last several years, circulating natriuretic peptides have become widely accepted as diagnostic and prognostic measures in all stages of CHF [5], [6], but also other complementary neurohumoral markers are intensively studied in this field [7], [8].

Increased circulating levels of ET-1 have been demonstrated in patients with CHF [3], [4], [9], [10], [11], but its prognostic significance has remained controversial so far. There is some evidence that high circulating ET-1 may be linked to poor outcomes in these patients [4], [9], [10], particularly in those at advanced stages of the disease [3], however, these associations have not been confirmed by all groups of researchers [6], [12], [13]. Unequivocal results of high plasma ET-1 levels and increased mortality in patients with CHF may be due to difficulties in the precise measurements of the rather unstable ET-1 in circulating blood (resulting from its short plasma half-life [14], its intermediate clearance by receptor binding in the pulmonary vascular bed [15], and its degradation by neutral endopeptidases [16], [17]. An alternative measure of the activity of the ET-1 system may be the assessment of circulating C-terminal pro-endothelin-1 (CT-proET-1), the C-terminal endothelin-1 precursor fragment, which is more stable than the active molecule and presumably inactive in the circulation [18], [19].

We hypothesized that measurement of plasma CT-proET-1 would carry additional prognostic value in a population of patients with CHF, beyond other generally accepted prognostic markers, including clinical measures of CHF severity and plasma N-terminal pro-B-type natriuretic peptide (NT-proBNP).

## Methods

### Study Population

We tested the aforementioned hypothesis in 491 patients with systolic CHF enrolled at 4 European centers: Verona (Italy, n = 230, 47%), Wroclaw (Poland, n = 122, 25%), London (United Kingdom, n = 80, 16%), and Athens (Greece, n = 59, 12%). The criteria for inclusion in our study were: 1) a ≥3 months documented history of CHF; 2) left ventricular ejection fraction (LVEF) ≤45% as assessed by echocardiography; 3) clinical stability and unchanged medications for at least one month preceding the study. The following exclusion criteria applied: a) revascularisation and/or acute coronary syndrome within 3 months preceding the study; b) circulatory decompensation within 1 month preceding the study; 3) lack of informed written consent. Inclusion and exclusion criteria were uniform in all participating centers.

In 11 additional cases (not included in the analyses), plasma CT-proET-1 levels were not available for technical reasons. Clinical characteristics of all studied patients are presented in Table 1. The study protocol was approved by the local ethics committee and all subjects gave written informed consent. The study was conducted in accordance with the Declaration of Helsinki.

**Table 1:** Clinical characteristics of patients with CHF.

Variables	Patients with CHF (n = 491)
Age, years	63±11
Sex, men/women	91% / 9%
BMI, kg/m^2^	26.3±4.1
NYHA class, I/II/III/IV	9% / 45% / 38% / 8%
CHF etiology, CAD/non-CAD	69% / 31%
LVEF, %	31±8
Hemoglobin, g/dL	13.8±1.6
Serum creatinine, µmol/L	100 (87–126)
Plasma CT-proET-1, pmol/L	63.6 (49.5–87.1)
Plasma NT-proBNP, pg/mL	875 (347–2465)
Treatment:	
ACE inhibitor and/or angiotensin receptor blocker	89%
β-blocker	61%
loop diuretic	78%
spironolactone	245%
digoxin	37%
statin	53%
acetylsalicylic acid	57%

Data are presented as mean±standard deviation, median with interquartile range or n (%) where appropriate. BMI – body mass index, NYHA – New York Heart Association, CHF –chronic heart failure, CAD – coronary artery disease, LVEF – left ventricle ejection fraction, CT-proET-1 – C-terminal pro-endothelin-1, NT-proBNP – N-terminal pro-B-type natriuretic peptide, ACE – angiotensin converting enzyme.

### Plasma CT-proET-1 and NT-proBNP Measurements

In all patients with CHF, venous blood samples were taken in the morning following an overnight fast and after supine rest of at least 15 min. After centrifugation, plasma was collected and frozen at −80°C until being analysed. Plasma levels of CT-proET-1 (pmol/L) were assessed using a chemiluminescence sandwich immunoassay that used 2 polyclonal antibodies to amino acids 168–212 of pre-proET-1 (CT-proET-1 LIA, B.R.A.H.M.S GmbH, Hennigsdorf/Berlin, Germany) as previously described [16]. The analytical detection limit for plasma CT-proET-1 is 0.4 pmol/L. The interlaboratory variability coefficient is <10% for values >10 pmol/L [17], [18]. The stability of CT-proET-1 in plasma at room temperature is at least 4 hours [18].

Plasma levels of NT-proBNP (pg/mL) were determined by an electrochemiluminescence immunoassay on the Elecsys 1010/2010 System (Roche Diagnostics GmbH, Mannheim, Germany).

### Clinical Follow-up

Patients were regularly seen by the study investigators with a follow-up duration of at least 12 months in all who survived. Information regarding survival was obtained directly from patients or their relatives, from the CHF clinic database, or from the hospital system. No patient was lost to follow-up. The primary end-point for the analysis was 12-month cardiovascular mortality. Cardiovascular mortality was defined as death due to myocardial infarction, sudden cardiac death, due to the progression of CHF, or death related to strokes.

### Statistical Analyses

Continuous variables were expressed as mean ± standard deviation (SD). The inter-group differences were tested using Student's t-test, the χ^2^ test, or one-way ANOVA as appropriate. Plasma CT-proET-1, NT-proBNP and serum creatinine had skewed distributions and were therefore log_10_ transformed before analyses. They were expressed as medians with interquartile ranges (IQR). The inter-group differences were tested using the Mann-Whitney U test, the Pearson χ^2^ test, or the Kruskal-Wallis test as appropriate. Spearman's rank correlation and multivariable regression analyses using ordinary least squares were applied to establish variables plasma CT-proET-1 levels in patients with CHF.

The associations between analysed variables and survival were assessed using Cox proportional hazards analysis (both single predictor and multivariable models). In the multivariable analyses, we included age, New York Heart Association (NYHA) class, LVEF, and creatinine, as well as plasma levels of neurohormonal markers (CT-proET-1, NT-proBNP). NYHA class was included as a categorized variable. Due to the low number of patients in NYHA class I and a similar mortality rate in NYHA classes I and II, both groups were combined and were used as reference group. The assumptions of proportional hazards were tested for all the covariates. For continuous variables, hazard ratios (HR) per interquartile range (IQR) are reported (standardized HR). 95% confidence intervals (CI) for risk factors and significance levels for χ^2^ (Wald test) are given. The predictive value of each model was assessed by the model likelihood ratio χ^2^ statistic. In addition, to describe and compare the predictive performance of each multivariable model, the concordance index (c index) was calculated using a Harrel's method [19], [20]. To penalize over-fitting by entering multiple factors, the bootstrap-corrected version of the c index is given. Plasma levels of both CT-proET-1, NT-proBNP and serum creatinine were included in the model after log_10_ transformation.

In order to illustrate the effect of plasma CT-proET-1 on 12-month cardiovascular mortality rates, Kaplan-Meier curves for cumulative survival were constructed. For continuous variables, subsequent quartiles were used. Differences in survival rates were tested using the Cox-Mantel log-rank test. Receiver-operating characteristic (ROC) curves for prediction of 12-month mortality were constructed and the area under the curve (AUC) with a 95% confidence interval were calculated. To further contrast the prognostic accuracy of biomarkers, statistical comparison of ROC curves was performed using the method of paired ROC curves described by Hanley and McNeil [21]. A value of p<0.05 was considered statistically significant. Statistical analyses were performed using StatView 5.0 for Windows (Abacus Concepts, Berkley, CA) and R version 2.5.1 (http://www.r-project.org). All authors had full access to the data and took responsibility for the integrity and accuracy of the analyses.

## Results

Plasma levels of CT-proET1 were evaluated in 491 patients with systolic CHF. For the additional 11 samples (all survivors), valid CT-proET-1 levels could not be determined due to a complete degradation. The median level of plasma CT-proET-1 was 63.6 pmol/L (IQR: 49.5–87.1 pmol/L).

### Relationships Between Plasma CT-proET-1 and Clinical Variables in Patients with CHF

**Figure 1:**
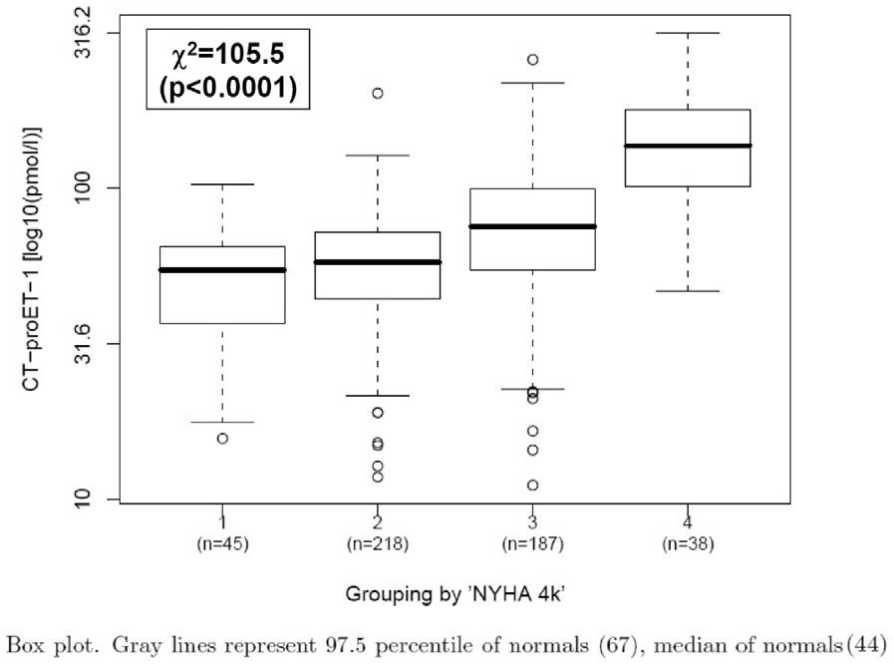
Medians and interquartile range of plasma CT-proET-1 in 491 patients with CHF according to NYHA class.

Median levels of plasma CT-proET-1 increased with increasing NYHA class (Figure 1). In single predictor regression analyses, plasma CT-proET-1 (log_10_) correlated positively with age (r = 0.33), serum creatinine (log_10_, r = 0.40) and plasma NT-proBNP (log_10_, r = 0.44, all p<0.0001). In single predictor regression analyses, plasma CT-proET-1 (log_10_) was inversely related to hemoglobin level (r = −0.26, p<0.0001), BMI (r = −0.12, p = 0.012) and LVEF (r = −0.15, p = 0.0013). Higher plasma CT-proET-1 levels were found in patients with ischemic CHF in comparison to subjects with non-ischemic CHF (p = 0.0002).

In a multivariable regression model based on all parameters included in univariate models, plasma CT-proET-1 (log_10_) was related significantly to NYHA class (r = 0.26, p<0.0001), serum creatinine (log_10_, r = 0.21, p<0.0001) and plasma NT-proBNP (log_10_, r = 0.13, p = 0.0009). The model explained 32.9% (adjusted R^2^) of total variance of plasma CT-proET-1 in patients with CHF.

Patients with CHF were divided into 4 subgroups according to ascending quartiles of plasma CT-proET-1, and clinical characteristics of these subgroups are presented in Table 2. Patients with CHF with the highest plasma CT-proET-1 (>upper quartile) had particularly high plasma NT-proBNP as compared to the 3 other subgroups and were characterised by unfavourable clinical features. Namely, they were older, had lower BMI, LVEF and hemoglobin levels, and higher NYHA class and serum creatinine levels. Moreover, they were more likely to have ischemic CHF (Table 2).

**Table 2:** Clinical characteristics of patients with CHF according to ascending quartiles of plasma CT-proET-1.

Variables	Q1: plasma CT-proET-1≤49.5 pmol/L	Q2: 49.5 pmol/L<plasma CT-proET-1≤63.6 pmol/L	Q3: 63.6 pmol/L<plasma CT-proET-1≤87.1 pmol/L	Q4: plasma CT-proET-1>87.1 pmol/L	Kruskal-Wallis x^2^ (KW), or Pearson x^2^ (P), p
Age, years	59±11	61±11	65±9	68±10	KW x^2^ = 54.5 (p<0.0001)
Sex, men	122 (99%)	110 (89%)	108 (89%)	109 (89%)	P x^2^ = 12.6 (p = 0.005)
BMI, kg/m^2^	26.8±4.1	26.1±4.1	26.8±4.0	25.3±3.9	KW x^2^ = 11.0 (p = 0.01)
NYHA class, III–IV	34 (28%)	39 (32%)	49 (40%)	103 (84%)	P x^2^ = 124.6 (p = 0.0005)
CHF etiology, CAD	71 (58%)	85 (69%)	84 (69%)	99 (80%)	P x^2^ = 13.97 (p = 0.003)
LVEF, %	31±9	32±8	31±8	28±8	KW x^2^ = 15.0 (p = 0.002)
Hemoglobin, g/dL	14.2±1.1	14.0±1.3	13.5±1.6	13.2±1.9	KW x^2^ = 29.4 (p<0.0001)
Serum creatinine, µmol/L	94 (82–107)	93 (84–105)	107 (94–128)	131 (96–158)	KW x^2^ = 79.2 (p<0.0001)
Plasma NT-proBNP, pg/mL	497 (238–1245)	475 (229–1140)	861 (364–2123)	3110 (1120–6880)	x^2^ = 114.1 (p<0.0001)

Data are presented as mean±standard deviation, median with interquartile ranges, or n (%) where appropriate. BMI – body mass index, NYHA – New York Heart Association, CHF –chronic heart failure, CAD – coronary artery disease, LVEF – left ventricle ejection fraction, CT-proET-1 – C-terminal pro-endothelin-1, NT-proBNP – N-terminal pro-B-type natriuretic peptide.

### Survival Analyses

All survivors were clinically followed for up to 12 months. During follow-up there were 70 (14%) deaths, and 12- month mortality rate was 14%,. Mean time to death was 139±110 days (median: 116 days, range: 1–360 days). The proportionality assumption and the assumption of a log-linear relationship between the prognosticators and the hazard function were fulfilled for all tested variables.

#### Single Predictor Cox Regressions

In single predictor Cox proportional-hazards models, the following variables were related to increased 12-month cardiovascular mortality in patients with CHF (Table 3): high plasma CT-proET-1 (standardized HR = 2.4, p<0.0001), high plasma NT-proBNP (standardized HR = 3.36, p<0.0001), advanced NYHA class (both when categorised into 3 and 4 groups, p<0.0001), high serum creatinine (standardized HR = 1.58, p = 0.0019), and the following variables were related to reduced 12-month cardiovascular mortality in this group of patients: high LVEF (HR = 0.32, p<0.0001), increased hemoglobin level (standardized HR = 0.60, p = 0.0006) and increased BMI (standardized HR = 0.652, p = 0.02), but neither age, sex nor CHF etiology (all p>0.1).

**Table 3:** Predictors of 12-month cardiovascular mortality in patients with CHF (single predictor and multivariable Cox proportional hazard models).

Prognosticators (units)	Single predictor model		Multivariable model	
	HR (95% CI)	LRT p	HR (95% CI)	Wald p
Plasma CT-proET-1 (log)	2.41 (1.87–3.1)	<0.0001	1.42 (1.04–1.95)	0.0267
Plasma NT-proBNP (log)	3.36 (2.4–4.71)	<0.0001	1.57 (1.0–2.46)	0.0489
NYHA class		<0.0001		0.0358
III vs. I,II	3.25 (1.8–5.88)		2.03 (1.06–3.89)	
IV vs. I,II	10.9 (5.59–21.2)		3.06 (1.27–7.34)	
LVEF	0.321 (0.229–0.452)	<0.0001	0.40 (0.268–0.597)	<0.0001
Age	1.17 (0.862–1.59)	0.31	0.96 (0.69–1.34)	0.81
Serum creatinine (log)	1.58 (1.2–2.09)	0.0019	0.95 (0.67–1.34)	0.76
Hemoglobin	0.595 (0.445–0.795)	0.0006		
BMI	0.652 (0.448–0.947)	0.02		

For continuous variables standardized HR (HR per IQR increase) are calculated.

CI – confidence interval, LRT p – likelihood-ratio test p-value, CT-proET-1 – C-terminal pro-endothelin-1, NT-proBNP – N-terminal pro-B-type natriuretic peptide, BMI – body mass index, NYHA – New York Heart Association, CHF – chronic heart failure, CAD – coronary artery disease, LVEF – left ventricle ejection fraction.

**Figure 2:**
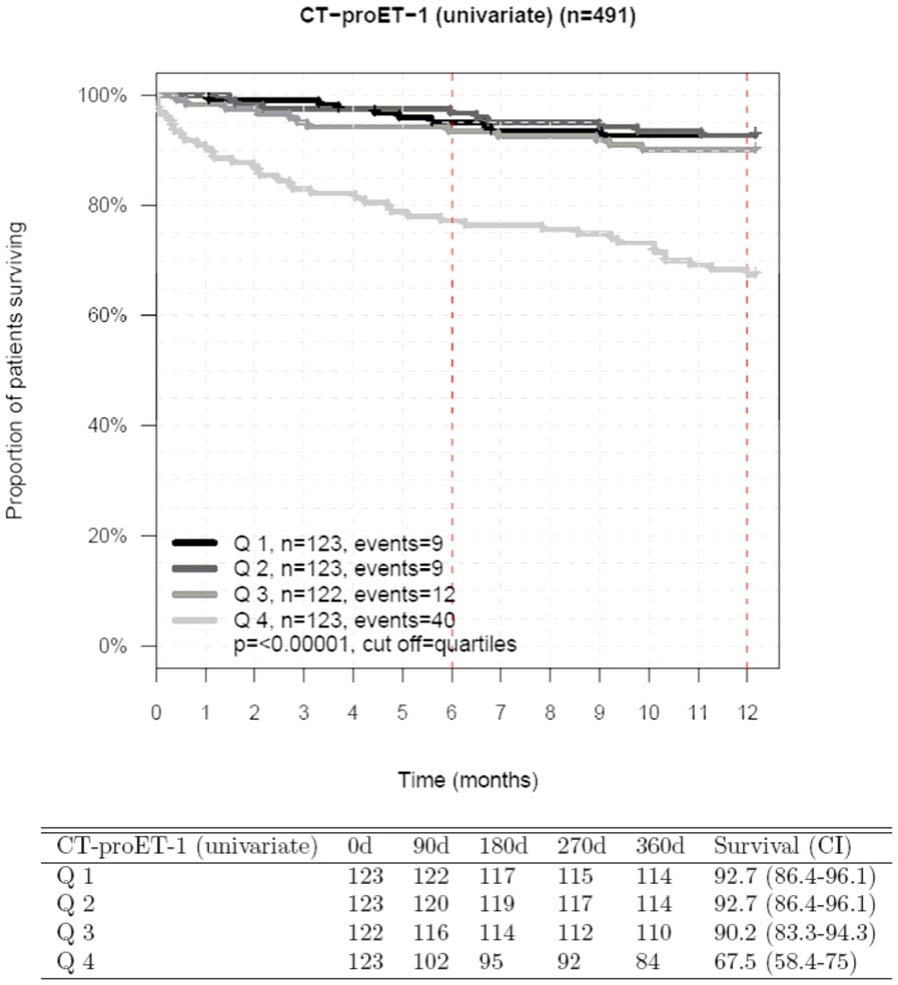
12-month cumulative survival rates in 491 patients with CHF divided according to ascending quartiles of plasma CT-proET-1.

Twelve-month survival rates were 93% (95% CI: 86–96%), 93% (95% CI: 86–96%), 90% (95% CI: 83–93%) and 68% (95% CI: 58–75%) for those patients with plasma CT-proET-1 in ascending quartiles (Figure 2). Whilst patients with plasma CT-proET-1>87.1 pmol/L (upper quartile) had a particularly high 12-month mortality (32%) as compared to the 3 remaining subgroups (12-month mortality: approximately 7–10%) (Figure 2).

In receiver operating characteristic curve analysis, areas under curve for 12-month follow-up were similar for plasma CT-proET-1 and plasma NT-proBNP (AUC = 0.724 (CI 0.682–0.763) and 0.751 (CI 0.710–0.788), respectively; p>0.4).

#### Multivariable Cox Regressions

In multivariable models that included LVEF, NYHA, age, NT-proBNP and creatinine, high plasma CT-proET-1 remained an independent predictor of increased 12-month cardiovascular mortality in patients with CHF (Table 3, p = 0.03). The higher the number of identified risk factors (plasma CT-proET-1>87.1 pmol/L [upper quartile], plasma NT-proBNP>2465 pg/mL [upper quartile], NYHA class III–IV, LVEF≤31% [median]), the worse the prognosis (risk factor model, Table 3).

#### Comparison of the Predictive value of CT-proET-1 and NT-proBNP

The predictive values of different models were evaluated using the likelihood ratio χ^2^ statistic. A model adjusted for LVEF, age, serum creatinine, and NYHA class was defined as a base model. This model was compared with models including CT-proET-1, NT-proBNP or both biomarkers. Using the likelihood ratio χ^2^ test for nested models, CT-proET-1 and NT-proBNP were shown to add a significant prognostic value to the base model (p = 0.0003 and 0.004, respectively). CT-proET-1 added prognostic value to the base model including NT-proBNP (p = 0.02), and NT-proBNP added predictive value to a model that includes the variables of the base model plus CT-proET-1 (p = 0.045) (Table 4).

**Table 4:** Comparison of predictive power of multivariable models including plasma NT-proBNP and/or CT-proET1 in patients with CHF.

*Multivariable model*	χ^2^	d.f.	p	C index	Gain to base model (p)
Base model: LVEF, NYHA class, age, serum creatinine	77.0	5	<0.0001	0.766	-
Base model, plus plasma NT-proBNP	85.4	6	<0.0001	0.780	0.0038
Base model, plus plasma CT-proET-1	90.2	6	<0.0001	0.774	0.0003
Base model, plus plasma NT-proBNP and CT-proET-1	94.3	7	<0.0001	0.785	0.0002

CI – confidence interval, p – likelihood-ratio test p-value, CT-proET-1 – C-terminal pro-endothelin-1, NT-proBNP – N-terminal pro-B-type natriuretic peptide, NYHA – New York Heart Association, CHF – chronic heart failure, LVEF – left ventricle ejection fraction. C index – bootstrap-corrected concordance index.

## Discussion

Here we demonstrate that high plasma CT-proET-1 predicts increased 12-month cardiovascular mortality in patients with CHF, independently of clinical disease severity and plasma NT-proBNP.

The ET-1 system plays a crucial role in the pathophysiology of CHF [1], [2], [22]. Both vasoconstriction with unfavourable haemodynamic effects and cardiovascular remodelling contribute to the development and progression of CHF [1], [2], [22]. Recently, a new technique for the measurement of CT-proET-1, a stable ET-1 precursor fragment, has been developed [23]. CT-proET-1 has been used in several patient populations including patients with acute shortness of breath presenting to the emergency department [23], [24] and in stable outpatients with CHF [25], [26], [27]. Our study confirms and extends these studies, and we have demonstrated that plasma levels of CT-proET-1 are increased in patients with CHF as compared to values found in healthy controls in a previous study (mean: 44.3 pmol/L, range: 10.5–77.4 pmol/L) [28]. In patients with CHF, plasma CT-proET-1 correlates positively with disease severity (NYHA class). Moreover, patients with high plasma CT-proET-1 are older, have reduced hemoglobin levels and impaired renal function. These findings are analogous to the results obtained with regard to plasma ET-1 that were also increased in patients with CHF [3], [4], [9], [10], [11] and were positively associated with NYHA class [3], [9], [10], [11]. Similarly, big ET-1, a precursor of ET-1, is present in excess in the peripheral circulation in patients with CHF, particularly in advanced stages of disease [3], [27], [28]–[32].

So far, the prognostic value of plasma CT-proET-1 measurements has been proven only in patients during the acute phase of myocardial infarction [33], [34]. High plasma CT-proET-1 together with plasma NT-proBNP predicts an unfavourable outcome during the follow-up of 12 months [33]. In the current analyses, we have extended the prognostic applicability of plasma CT-proET-1 measurements to patients with systolic CHF, irrespective of etiology and disease severity. Some authors have demonstrated increased mortality in subjects with high plasma ET-1 in a whole population of patients with CHF [4], [9], [10] or in those with advanced CHF (NYHA III–IV) [3], but the other authors failed to confirm these associations [32], [33], [34]. However, all available data confirm the existence of relationships between high plasma big-ET-1 and poor outcome in patients with CHF [27], [28], [29], [31], also in those with severe CHF [3], [30], [32]. Therefore, we suggest that an assessment of ET-1 precursors (big ET-1 or CT-proET-1) rather than an active final molecule ET-1 may provide useful prognostic information in patients with CHF.

In some analyses, high plasma big ET-1 [27], [30], [32] or high plasma ET-1 [4], [9] was a better discriminator in predicting death in patients with CHF than plasma levels of natriuretic peptides, particularly in advanced stages of the disease [27], [30], . On the other hand, some workers have confirmed the superiority of circulating natriuretic peptides over plasma big ET-1 or ET-1 in survival analyses in patients with mild to moderate CHF [11], [13], [32] and severe CHF [11], [12], [28]. One limitation of the aforementioned studies may be the fact that relatively small groups of patients with CHF were examined. The most comprehensive analysis was performed in 2359 patients with CHF, enrolled in the Valsartan Heart Failure Trial (Val-HeFT) [29]. In this cohort, in multivariable survival analyses both plasma big ET-1 and plasma BNP (brain natriuretic peptide) were related to mortality, and combined mortality and morbidity (median follow-up: 23 months) [29]. Whilst plasma BNP was the stronger prognostic marker, the inclusion of plasma big ET-1 into the model increased the predictive power of the whole set of prognostic variables [29]. In the present analyses, plasma levels of both CT-proET-1 and NT-proBNP significantly and independently of each other predicted an increased 12-month cardiovascular mortality in patients with CHF, but the association between plasma CT-proET-1 and 12-month prognosis was stronger. Moreover, adding plasma CT-proET-1 to the base model had stronger prognostic power than adding plasma NT-proBNP. Finally, adding plasma CT-proET-1 to plasma NT-proBNP in this model provided further prognostic information.

Some limitations of this analysis deserve discussion. First, the study population consisted mainly of male patients, and it is unknown whether these findings can be directly extrapolated to female patients. Moreover, the number of patients in NYHA class I was relatively small (9%) and novel surrogate biomarkers usually perform better in high-risk populations than in low-risk subjects or unselected populations. Finally, the study group represents a population with a high risk of cardiovascular events. These facts have to be kept in mind when the data are being interpreted.

### Conclusions

Plasma CT-proET-1 constitutes a novel predictor of increased 12-month cardiovascular mortality in patients with CHF, independently of disease severity and plasma NT-proBNP.
